# Alterations in the programming of energy metabolism in adolescents with background exposure to dioxins, dl-PCBs and PBDEs

**DOI:** 10.1371/journal.pone.0184006

**Published:** 2017-09-12

**Authors:** Marike M. Leijs, Janna G. Koppe, Thomas Vulsma, Kees Olie, Wim M. C. van Aalderen, Pim de Voogt, Juliette Legler, Gavin W. ten Tusscher

**Affiliations:** 1 Department of Paediatrics and Neonatology, Emma Children’s Hospital Academic Medical Center, Amsterdam, The Netherlands; 2 IBED/AEE, University of Amsterdam, Amsterdam, The Netherlands; 3 Ecobaby Foundation, Loenersloot, The Netherlands; 4 KWR Watercycle Research Institute, Nieuwegein, The Netherlands; 5 Institute for Environmental Studies (IVM) VU University, Amsterdam, The Netherlands; 6 Department of Paediatrics and Neonatology, Westfriesgasthuis, Hoorn, The Netherlands; Centre Hospitalier Universitaire Vaudois, FRANCE

## Abstract

**Objectives:**

Dioxins and PCBs are highly toxic and persistent environmental pollutants that are measurable in humans worldwide. These persistent organic pollutants are associated with a higher incidence of diabetes mellitus. We hypothesise that perinatal (background) exposure to industrial pollutants like dioxins also influences body mass development and energy metabolism in later life.

**Study design:**

In The Netherlands, the perinatal exposure (prenatal exposure and postnatal lactational intake) to dioxins has been studied prospectively since 1987. Fasting glucose, insulin, HbA1c and leptin were analysed in 33 children of the original cohort of 60. BMI, glucose:insulin and BMI:leptin ratios were calculated. Prenatal exposure, lactational intake and current serum levels of dioxins (PCDD/F), dl-PCBs and PBDE concentrations were determined using (HR)GC-MS.

**Results:**

Prenatal dioxin (PCDD/F) exposure was positively correlated to the glucose:insulin ratio (p = 0.024) and negatively correlated to the fasting insulin concentration (p = 0.017) in adolescence. Postnatal lactational PCDD/F intake was also negatively correlated to fasting insulin concentration (p = 0.028). Current serum levels of PCDD/Fs and total TEQ (dl-PCBs+PCDD/Fs) were positively correlated to the fasting serum glucose concentration (p = 0.015 and p = 0.037, respectively).No metabolic effects were seen in association with current serum levels of PBDEs. A positive correlation between the insulin and leptin concentrations (p = 0.034) was observed. No effects were found on leptin levels, BMI:leptin ratio, HbA1c levels or BMI.

**Discussion/Conclusion:**

This study indicates that prenatal and lactational exposure influences glucose metabolism in adolescents, presumably through a negative effect on insulin secretion by pancreatic beta cells. Additionally, the very low recent background exposure to dioxins in puberty possibly has an effect on the glucose level.

## Introduction

Polychlorinated dibenzo-*p*-dioxins (PCDDs), polychlorinated dibenzofurans (PCDFs) and polychlorinated biphenyls (PCBs) belong to the group of most hazardous environmental toxicants. PCDDs and PCDFs are unintentionally formed by-products of the incineration of waste, metallurgic processes, the production of chlorinated phenols, and bleaching of paper pulp [1, 2]. PCBs were intentionally produced mainly because of their extreme stability and resistance to acids, bases, hydrolysis and heat [3]. As a result of emissions during these processes, these compounds are widespread in nature and bio-accumulate, resulting in the contamination of human food and breast milk. As a result, all European children are exposed to detectable levels [4]. PCDDs, PCDFs and planar PCBs are grouped together as ‘dioxins’ or ‘dioxin-like compounds’, because of planar structure which gives the ability to bind the aryl hydrocarbon (Ah) receptor. Genomic and non-genomic pathways have been identified after binding the Ah-receptor. The genomic (or classical action) pathway is interrelated with the retinoic acid (RA) signalling pathway [5]. Binding with this receptor mediates many pathological processes like teratogenesis and tumor promotion as well as disturbances in many metabolic processes in humans and animals [6]. The non-genomic pathway has been identified to play an important role in the inflammatory action of dioxins (intracellular Ca^2+^ concentration, enzymatic activation of phospholipase A2 and Cox2) [7] as well as other processes like insulin secretion, fat storage, liver damage and tumorigenesis, and is potentially regulated by miRNAs [8]. Dioxin-like compounds as well as PCBs have been proven to be endocrine disrupters by a variety of mechanisms [9–11].

Other, more recently produced persistent toxic compounds are the flame retardants, polybrominated diphenylethers (PBDEs) [12]. An increase in serum concentrations of these compounds began in the nineties [13]. Over the last three decades these compounds have been used more and more frequently in various materials such as electronic equipment, plastics, carpet liners and furniture textiles. Human uptake is mostly through ingestion by eating polluted food. Inhalation of dust is also an important exposure route, especially in younger children [14].

Diabetes and obesity are currently epidemics throughout the Western World. Besides eating habits and genetic factors, environmental factors such as smoking during pregnancy and famine during the first three months of pregnancy, followed by an abundance of food in utero and later, play a role in later body mass index (BMI) and energy metabolism [15]. Various correlations between persistent organic pollutants, especially dioxins, and insulin resistance and diabetes mellitus have been published [16].

The environment in early (perinatal) life sets the stage for lifelong health. There is empirical support from the Dutch famine study that early-life environmental conditions can cause epigenetic changes that persist throughout life [17].

Most studies on dioxins in relation to diabetes are based on cohorts of highly exposed populations, such as veterans exposed to dioxins through contact with the defoliant Agent Orange [18;19], and the highly exposed Yucheng cohort, exposed to PCBs and PCDFs through contaminated rice oil [20]. A higher incidence of diabetes was also seen in the Italian Seveso cohort, exposed to dioxins following an explosion at a herbicide manufacturing plant [21] and in a recent German occupational study [22]. Studies of lower exposed subjects include studies from Japan [23], the National Health and Nutrition Survey from the US [24], and Belgium. [25]. Most of these studies, however, were not prospective but cross sectional and the studies were performed in adults. Limited prospective studies have focussed on developmental exposures to dioxin, and to our knowledge, no epidemiological studies have been carried out which examine possible effects of PBDE exposure on diabetes and obesity.

Our prospective study of mother-baby pairs was started in 1987-1990/1991 to study the effects of dioxins in the prenatal and nursing period [26]. Previous results linked to metabolism include a significant decrease in serum retinol binding protein (RBP) found during the eleventh postnatal week in relation to a higher lactational exposure to dioxins and furans (PCDD/Fs). This finding is suggestive of an inhibiting effect of dioxins on adipocyte differentiation, that normally takes place after birth, since RBP is a protein produced by mature adipocytes [27]. Interestingly, the inhibition of adipocyte differentiation by TCDD has been shown in vitro [28].

These results indicate that dioxin exposure has biological effects early in life. Given the associations mentioned in the aforementioned studies, we analysed energy metabolism parameters in the cohort, including fasting glucose, fasting insulin, glucose:insulin ratio, glycosylated haemoglobin (HbA1c), and leptin, in relation to prenatal and lactational dioxin exposure as well as current dioxin, dioxin-like PCB and PBDE concentrations.

Leptin is a hormone produced by white adipose tissue. It has an important function in the regulation of appetite in the hypothalamic appetite centre, giving a feeling of satiation in order to limit caloric surplus. It also plays a role in the energy production from fatty acids in skeletal muscle cells [29].

We hypothesise that prenatal, lactational and current dioxin and PBDE exposure affects metabolic processes. Theoretically, this may lead to an increase in the risk of developing diabetes and obesity.

To our knowledge this is the first human study evaluating metabolic parameters after perinatal and current exposure in puberty to dioxins. In addition, effects of the current exposure to PBDEs were studied.

## Methods and materials

### Study population

In 1987 in the Amsterdam-Zaandam region a longitudinal cohort study on the effects of background exposure to dioxins was started. Selection criteria of the cohort were an optimal pregnancy, birth weight above 2500 grams, gestational age between 37 and 41 weeks and all the children were to be breast fed. This study is part of the longitudinal cohort study of 14–19 year old children, studied previously during their neonatal (n = 60) [30], toddler (n = 31) [31] and pre-pubertal period (n = 41)[32]. In the current (pubertal) follow-up all 33 children (18 girls and 15 boys) participating were born in the Amsterdam/Zaandam region and 25 of them are still inhabitants of this region. Prenatal PCDD/F exposure and lactational PCDD/F intake (together the perinatal exposure) were determined in breast milk soon after birth [33].

The participants and their parents were interviewed to obtain data on individual characteristics including residential histories, social economic status, smoking habits, past history of diseases and treatments as well as present current illnesses, medication usage and allergies. A physical examination by one and the same physician took place to assess the height and weight as well as the pubertal development [9]. Body mass index was calculated as weight (in kilograms) divided by height (in meters) squared.

Of the total cohort of 41 subjects who participated in the pre-pubertal study, one subject was excluded from the current follow-up because of an Ewing sarcoma, one was partly excluded because of an extra Y-chromosome, and one had passed away due to leukaemia. Five subjects declined to participate in the follow-up, two could not be traced. One of the children, who did not participate in the pre-pubertal follow-up, consented to the current follow up. Of the 33 examined adolescents, 3 refused blood sampling.

The study was approved by the Medical Ethics Committee of the Academic Medical Centre, Meibergdreef 9, Amsterdam, and was conducted according to the principles expressed in the Declaration of Helsinki, and its amendments. Following verbal consent, written informed consent was obtained from all the children and their parents, prior to inclusion in the previous and current study.

### Laboratory analyses

For measuring fasting glucose, HbA1c, insulin, leptin, and PCDD/Fs, dioxin-like-PCBs (dl-PCB) and PBDE serum concentrations, 30 subjects underwent vena puncture in 2005/2006, following overnight fasting for at least six hours. Serum was obtained and stored at -20° C until analysis.

The serum leptin concentrations were analysed using radioimmunoassay (RIA) from Millipore in the department for clinical chemistry in the VUMC in Amsterdam. Insulin samples were measured using the Immunoradiometric assay (IRMA) technique from Biosense in the Algemeen Medisch Laboratorium (AML) in Antwerpen. Glucose was determined using the hexokinase method and HbA1c using HPLC in the department for clinical chemistry in the Zaans Medisch Centrum in Zaandam. Perinatal PCDD/F levels and recent serum levels of PCDD/Fs, dl-PCBs and PBDEs were measured in an uncontaminated laboratory dedicated to low-level dioxin sample treatment, at the Environmental Chemistry Section of IBED/ESS of the University of Amsterdam. Levels of the 19 most toxic PCDD/F congeners (seven PCDDs and twelve PCDFs) and 3 dl-PCBs (77, 126, 169) as well as 8 PBDEs (28, 47, 85, 99, 100, 153, 154 and 183) were determined. The perinatal dioxin levels are expressed as International Toxic Equivalents (I-TEQ). The concentration of PCDD/F and dl-PCB congeners are expressed in TEQ pg/g lipid using WHO-TEF values [34].

For group separation of the compounds we used an activated carbon column (Carbosphere). The PCDD/F and dl-PCB fraction was isolated and a clean-up was performed using a column of AgNO_3_ -impregnated silica gel and a column of activated Al_2_O_3_ on top of silica gel. The PBDE fraction was purified using activated Al_2_O_3_ on silica gel and an activated alumina column. After concentrating the sample, quantification of dioxins and dl-PCBs was performed using HR-GC/HR-MS. PBDEs were determined using HR-GC/LR-MS. As an internal standard, a mixture of ^13^C-labelled PCDD/Fs, dl-PCBs and PBDEs was used. More detailed information about the analysis has been published previously [4].

PCDD/F concentrations were previously determined in mothers’ milk 3–4 weeks after birth, which is indicative of the *prenatal* exposure. The cumulative total *postnatal*/lactational intake was calculated as the measured PCDD/F concentration in breast milk multiplied by the total breast milk intake [33].

### Statistical analyses

For statistical analyses, simple linear regression, and Spearman’s correlation coefficient (when the correlation was not typically linear in the scatter diagram or when there were outliers) was used using SPSS®.

As dependent values we used serum glucose, serum insulin, glucose:insulin ratio, leptin, BMI and BMI:leptin ratio and HbA1c. The prenatal, lactational, serum PCDD/Fs and the serum dl-PCBs, the total dioxin WHO-TEQ (PCDD/F+dl-PCB) and PBDE concentrations were the independent values using linear regression and Spearman’s correlation coefficient.

To quantify the relationship of two variables, for linear regression the regression coefficient B was used and for Spearman’s rho the Spearman’s correlation coefficient. To adjust for possible confounding factors the partial correlation coefficient was used. The level of significance was 5% (p = 0.05).

#### Confounders

Evaluation of variables or confounding factors (age, sex and BMI) was performed.

Gender, age and BMI were considered possible confounders. Due to the limited size of the cohort, ANOVA was not viable. We therefore assessed the dependency according to the formula:

$$\begin{array}{l} y=N0+N1x+N2z+N3xz \end{array}$$

whereby x is the independent, y is the dependent and z the possible confounder.

Gender versus insulin (p = 0.815), versus glucose (p = 0.133) and versus the glucose:insulin ratio (p = 0.460) were not significant. In other words, gender was not a confounder in the outcomes. Similarly, age (p = 0.063, p = 0.054 and p = 0.336, respectively) and BMI (p = 0.640, p = 0.035 and p = 0.066, respectively) were analysed as possible confounders.

## Results

Descriptive statistics of the cohort (age, BMI, prenatal dioxin exposure, lactational dioxin intake and, current serum dioxin, dl-PCB and PBDE levels) are presented in Table 1. The measured serum dioxin (PCDD/Fs) levels in the adolescents (current serum levels) were much lower than the prenatal exposure (levels in the mothers). There was no significant correlation between the current serum levels and the prenatal exposure levels nor with the lactational intake. Descriptive statistics of the measured metabolic parameters are given in Table 2. A summary of the results of the statistical analyses is given in Table 3.

**Table 1:** Descriptive statistics of the cohort and the cohort´s exposure to PCDD/Fs, dl- PCBs and PBDEs.

N = 33	Median	Mean	Range	95^th^—percentile
Age (years)	14.3	15.0	14.0–18.7	18.5
Prenatal PCDD/F exposure I-TEQ (pg/g lipid)	29.8	32.6	9.05–88.8	74.8
LactationalPCDD/Fexposure I-TEQ (ng)	45.9	66.9	4.34–279	239.1
BMI (kg/m^2^)	19.7	21.0	17.4–30.9	29.9
Current serum PCDD/F WHO I-TEQ (pg/g lipid)	1.6	2.2	0.4–6.1	6.1
Current serum dl-PCBs WHO I-TEQ (pg/g lipid)	1.8	2.2	0.04–7.8	7.3
Current serum PBDEs(ng/g lipid)	9.9	14	4.9–73.6	22.1

**Table 2:** Descriptive statistics of the cohort´s measured metabolic parameters.

N = 33	Median	Mean	Range	Standard Deviation
HbA1c (%)	5.6	5.5	5–6.1	0.24
Leptin (μg/l)	5.3	6.9	0.5–27.6	7.1
Glucose (mmol/l)	4.0	4.0	3.2–5.0	0.4
Insulin (mU/l)	9.2	9.7	4.4–16.1	3.2

**Table 3:** Summary of the statistical significance (p-values) of relationships between exposure and metabolic parameters of the cohort calculated with linear regression/multiple regression and Spearman’s 2-tailed correlation.

	*Prenatal PCDD/F expos*ure I-TEQ (pg/g lipid)			Postnatal Lactational PCDD/F intake I-TEQ (ng)			Current total dioxins in serum WHO I-TEQ(PCDDF+dl-PCB) (pg/g lipid)			Current serum PCDD/F WHO I-TEQ (pg/g lipid)			Current serum dl-PCBs WHO I-TEQ (pg/g lipid)			Current serum PBDEs (ng/g lipid)		
	Slope (neg/ pos)	Linear regres-sion p-value	Spear-man 2-tailed corre-lation	Slope (neg/ pos)	Linear regres-sion p-value	Spear-man 2-tailed corre-lation	Slope (neg/ pos)	Linear regres-sion p-value	Spear-man 2-tailed corre-lation	Slope (neg/ pos)	Linear regres-sion p-value	Spear-man 2-tailed corre-lation	Slope (neg/ pos)	Linear regres-sion p-value	Spear-man 2-tailed corre-lation	Slope (neg/ pos)	Linear regres-sion p-value	Spear-man 2-tailed corre-lation
Glucose	+	0.631	0.631	+	0.278	0.287	+	0.037^a^	0.037^a^	+	0.015^a^	0.015^a^	+	0.994	0.994	+	0.564	0.564
Insulin	-	0.017^a^	0.017^a^	-	0.028^a^	0.028^a^	-	0.592	0.592	-	0.114	0.114	-	0.916	0.916	-	0.494	0.494
Glucose:insulin ratio	+	0.024^a^	0.023^a^	+	0.095	0.095	+	0.577	0.588	+	0.983	0.998	+	0.811	0.819	+	0.633	0.640
Leptine (lin. regression)	-	0.138	0.138	-	0.228	0.228	-	0.337	0.337	-	0.139	0.139	-	0.630	0.630	-	0.584	0.584
BMI	-	0.920	0.650	+	0.177	0.983	+	0.916	0.916	+	0.951	0.914	-	0.788	0.474	-	0.735	0.852
HbA1c	+	0.427	0.427	-	0.857	0.857	+	0.577	0.916	+	0.426	0.426	-	0.185	0.185	-	0.471	0.471
Age	+	Not relevant	Not relevant	+	Not relevant	Not relevant	-	0.191	0.191	-	0.391	0.391	-	0.354	0.354	+	0.838	0.838

^a^values correspond to statistically significant correlations

All clinical laboratory measurements in the adolescents were within normal limits. Our population of 33 adolescents had heights, weights and BMIs within the normal range for Dutch adolescents. The mean BMI was 21 kg/m^2^. There were no children with obesity, but 3 children (9%) were overweight (BMI 25–29.9). Life style (diets, consumption of alcohol, cigarettes) as well as socio economic status had no influence on the dioxin, dl-PCB or PBDE levels. None of the participants had a known exposure to other toxicants.

### Glucose concentrations, HbA1c levels

**Fig 1:**
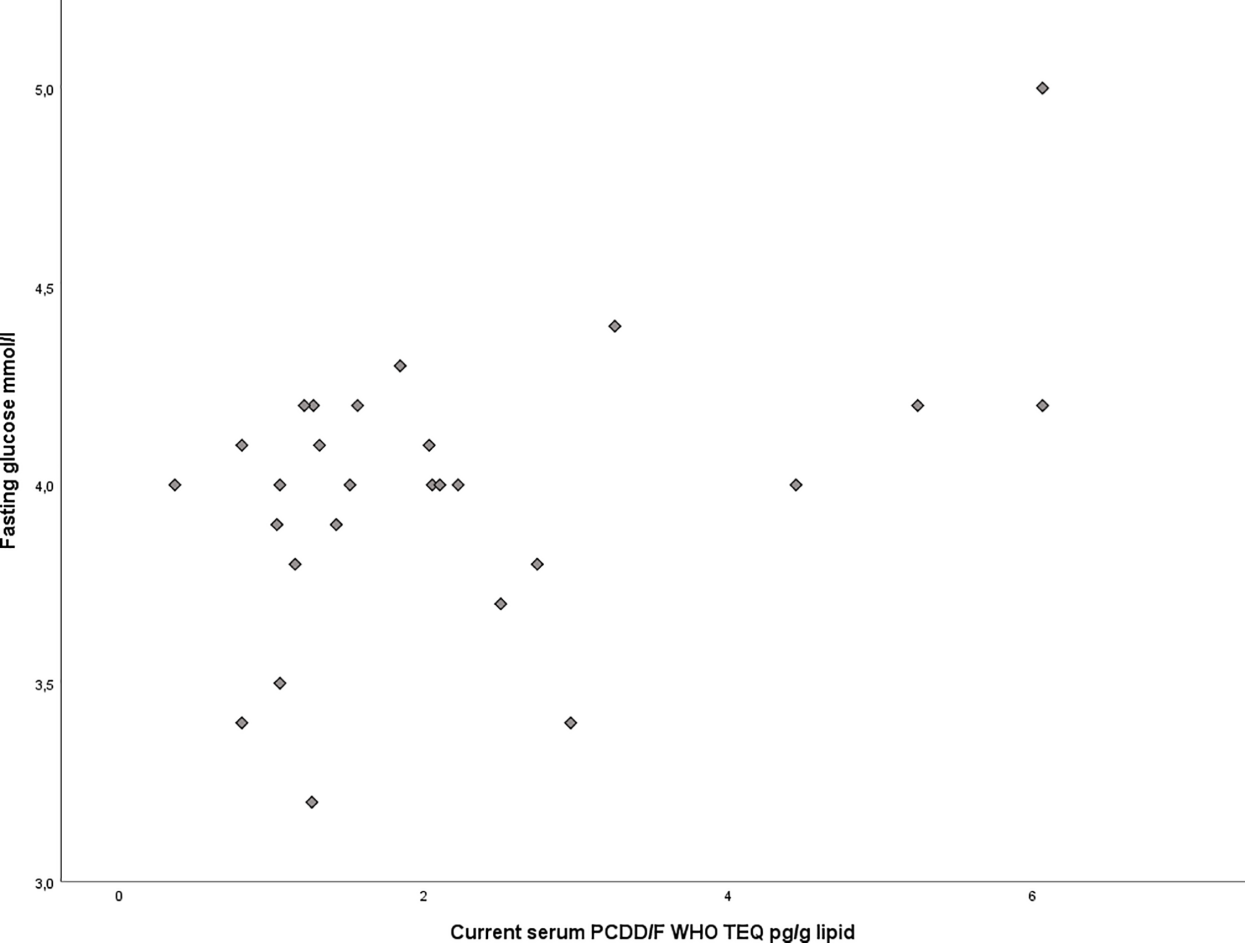
Current fasting glucose and current serum PCDD/Fs. (p = 0.015 correlation coefficient r = 0.46, std. error = 0.040).

Serum samples were taken to measure the fasting glucose in the adolescents. After statistical analysis using linear regression we found a significant correlation of glucose levels with current serum PCDD/Fs (p = 0.015, correlation coefficient (r) = 0.46, std. error = 0.040, see Fig 1). Neither gender, nor age or BMI were identified as confounding factors in this case.

We also found a significant positive correlation between the current total dioxin WHO-TEQ (PCDD/Fs+dl-PCBs) serum levels and fasting glucose concentrations (p = 0.037, correlation coefficient (r) = 0.43, std. error = 0.028). No correlation between dl-PCBs (p = 0.994), PBDEs (p = 0.564), lactational intake (p = 0.287) or prenatal (p = 0.631) PCDD/F exposures and fasting glucose levels were observed. Similarly, we found no correlation between HbA1c levels and PCDD/F, dl-PCB or PBDE serum levels, prenatal PCDD/F concentrations or lactational intake. HbA1c gives an indication of the average serum glucose concentration of the previous 2–3 months.

### Insulin concentration and glucose:Insulin ratio

**Fig 2:**
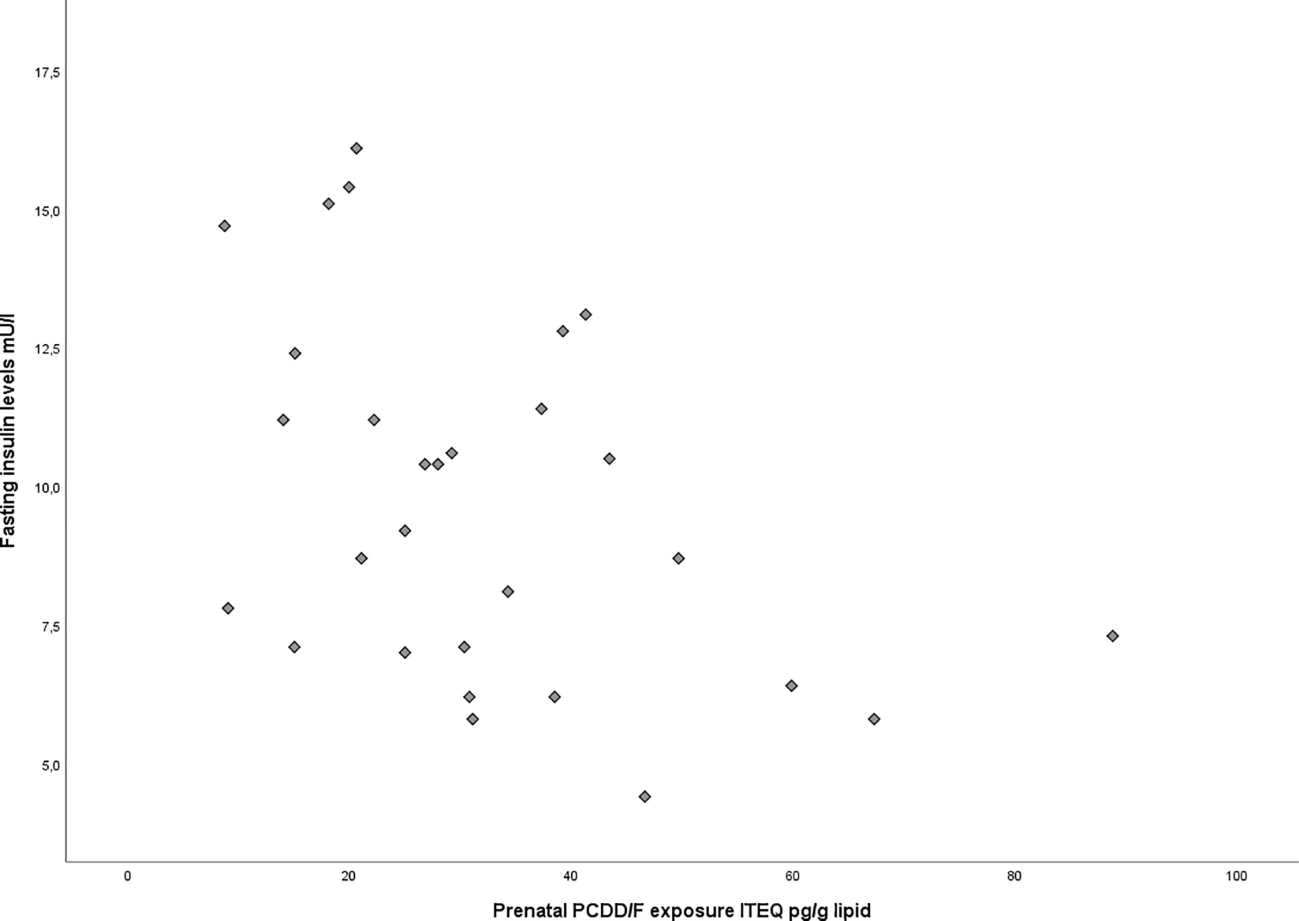
Fasting insulin levels and prenatal PCDD/F exposure. (p = 0.017, correlation coefficient r = -0.44, std. error = 0.031).

We observed a significant negative correlation between insulin levels and prenatal PCDD/F exposure (p = 0.017, correlation coefficient (r) = - 0.44, std. error = 0.031) (Fig 2), as well as with postnatal lactational intake (p = 0.028, (r = -0.41, std. error = 0.008). For the serum PCDD/F (p = 0.114) serum dl-PCBs (p = 0.916), and total WHO-TEQ in serum (p = 0.592), no correlation was seen with insulin concentrations. Age, sex nor BMI were identified as confounding factors in this model.

**Fig 3:**
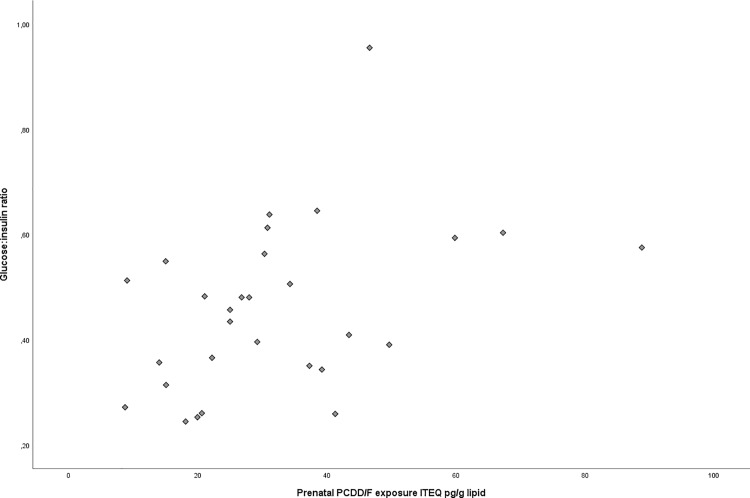
Glucose:insulin ratio and prenatal PCDD/F exposure. (p = 0.024, correlation coefficient r = 0.42, std. error = 0.002).

For the glucose:insulin ratio a significant positive correlation was seen with the prenatal PCDD/F exposure using linear regression (p = 0.024, r = 0.42, std. error = 0.002) (Fig 3). We found no correlation with postnatal lactational PCDD/F intake (p = 0.095) or current serum PCDD/F (p = 0.983), dl-PCB (p = 0.811) and PBDE (p = 0.633) levels.

### Body mass index

No significant correlation was observed between the current BMI and the prenatal exposure (p = 0.920) or lactational intake (p = 0.177) of dioxins, nor with the serum dioxin (p = 0.951), dl-PCB (p = 0.788) and PBDE (p = 0.735) levels. After separation of the group for boys and girls, no significant correlation was found for either group.

We found no correlation between the age of the group (p = 0.772) and the BMI.

### Leptin concentration and BMI:Leptin ratio

A clear positive correlation between BMI and serum leptin was found (p<0.001) using Pearson’s correlation coefficient. As expected, a positive correlation between leptin and insulin was also seen (p = 0.034). Using the partial correlation coefficient showed that gender and age did not influence these correlations.

We found no correlation between leptin and the measured compounds, statistical information is found in Table 3.

The statistical analysis of leptin concentrations in relation to the environmental pollutants was strongly influenced by the confounding factor gender. Analysis stratified by gender showed no significant correlations with the measured compounds (p = 0.979 for boys and p = 0.230 for girls). Similarly, no significant correlations were found for the BMI:leptin ratio and the measured compounds when using gender stratified analysis.

## Discussion

### Glucose metabolism

The main finding of this study is a negative correlation between (perinatal) dioxin exposure on glucose metabolism in adolescence. Lower insulin secretion in adolescence was seen in association with both prenatal exposure and lactational dioxin intake. The glucose:insulin ratio is positively associated with prenatal dioxin exposure. The very low serum PCDD/F levels (mean 2.2 pg/g fat) and total WHO- TEQ serum levels (PCDD/F+dl-PCB levels (mean 4.4 pg/g fat)) were positively correlated with serum fasting glucose levels.

We found no effects on serum glucose, insulin or the glucose:insulin ratio with serum PBDE levels in the adolescents.

Effects however, were seen in an animal study. One study found a higher glucose:insulin ratio in PBDE (DE-71) treated rats compared to controls. Fasting plasma glucose, insulin, and C-peptide levels were not markedly affected in this study [35]

We have to bear in mind that PBDE congeners have a much shorter half-life than dioxins. This means that a single measurement of serum levels might be insufficient to give an indication of long term exposure to these compounds.

Our results suggest an influence of dioxins in the perinatal period on pancreas development.

The significant correlation between current serum dioxin exposure and fasting serum glucose, confirms findings seen in a study with higher dioxin exposure [36], and a study of Agent Orange veterans exposed to the TCDD-containing defoliant [37], as well as the Seveso study [21]. Lower insulin levels were also found in a study of Faroese residents in relation to PCBs [38]. These findings in adult populations are also seen in our adolescent cohort, with the current very low background exposure. However contrasting studies have also been published. In a study on rat insulin-secreting beta cell lines, higher levels of insulin were observed after stimulation with TCDD. In this study a continuous insulin secretion was observed by stimulation of exocytosis for secretory vesicles containing insulin followed by beta cell exhaustion [39]. In a mice study, chronic exposure to Aroclor 1254 (a mixture of PCB congeners) induced hyperinsulinemia with an elevation of glucose and glucagon levels. Hereby it was observed, that Aroclor 1254 inhibited the expression of the insulin receptor signaling cascade [40].

In a cross-sectional study on Danish children, a reduced serum insulin was seen with higher PCB levels. However, in the study no separate statistical analyses were made on dl- and non-dl-PCB congeners, though different congeners may exert different effects. No effect on fasting glucose was seen in the study [41].

We found no correlation between HbA1c levels and PCDD/Fs, dl/PCBs or PBDEs. This could be due to the limited number of studied subjects or the low exposure status of the cohort. One cross-sectional study with 1374 (15–73 y) participants (mean total TEQ 24.08) in Japan found a correlation between HbA1c and the total TEQ of PCDD/Fs and dl-PCBs [23].

Direct effects of dioxins on insulin secretion have been described in animal studies. The disruptive effect of dioxins on beta cell functioning and lower insulin secretion of pancreatic islands was seen in a rat study [42]. In a study in mice, the insulin secretion was found to be significantly decreased by TCDD exposure, probably via the AhR signaling pathway[43].

Another example of disturbances of the energy homeostasis after dioxin exposure is the commonly seen wasting syndrome. This is known as the acute state of (permanent) reduced food intake and wasting after acute dioxin poisoning. Although the exact mechanism of this syndrome is unknown, there are signs of derangement of the energy balance [44].

Another explanation for our findings, the prenatal and lactational exposure to dioxins and later effects on insulin secretion might be caused by epigenetic changes. There is empirical support that early-life environmental conditions can cause epigenetic changes that persist throughout life [17].

Dioxins have been shown to exert epigenetic effects in sperm cells [45;46].

Possible epigenetic changes could involve the SOX9 gene [47]. Its homolog in zebra fish is downregulated by dioxins. [48]. Both SOX 9 and PDX1 genes, important for the development of the beta cells, are two of the earliest genes expressed in pancreatic tissue [49]. An association between increased methylation of the promoter of the RXRA gene in the cord blood of children, and later obesity was seen in 2 cohorts in the UK [50].

Theoretically, the perinatal dioxin exposure, that was quite high in Western Europe when our cohort was born (1987–1991), could have left modifications in the form of epigenetic changes resulting in effects on glucose metabolism in later life.

### BMI and leptin

No correlation was seen between BMI and the perinatal dioxin exposure, including after correction for confounding factors (age, gender). No correction was made for the confounding factor parental BMI, however the mothers’ BMI was divided equally in two different groups and there were no obese women who participated the study [51]. It must be borne in mind that the number of subjects in the study is limited, and the children at the time of blood sampling, were still in their puberty, a period with changing BMIs. The adolescents now studied had no history of severe illnesses, and no abnormal weight gain was observed.

We could find one animal TCDD study that reports on serum concentrations of leptin, glucose, insulin and triglyceride in rats. In this study, similarly to our study, no effects on leptin concentrations were observed [44]. In a recent Japanese study, serum levels of leptin were significantly lower in the highly (PCB/PCDF) exposed Yusho victims [52].

A limitation of our present study is the rather small number of participants. Furthermore, the fact that since only breast fed participants were included, we cannot exclude modification by breast feeding. The HOMA-IR and HOMA- β was not calculated in this study. The effect of exposure to other compounds, like DDT (dichlorodiphenyltrichloroethane), hexachlorobenzene, non-dioxin-like PCBs, PBB (polybrominated biphenyls), perfluorooctane sulfonate, phtalates and bisphenol-A cannot be excluded.

Further research on the possible mechanism and interaction of dioxins with the developing pancreas and effects at older age is warranted.

## Conclusion

Effects seen in this prospective long-term study of mother-baby pairs, selected on the basis of an optimal pregnancy, delivery and birth weight, indicate the influence of prenatal dioxin exposure on the metabolic parameter insulin secretion in later life. Since the levels of dioxin exposure are so-called background levels in Western Europe in the period 1987–1991, these findings warrant concern. Further follow-up needs to elucidate if these disturbances predict pathology, such as diabetes mellitus, in later life.

## Supporting information

S1 File Dataset SPSS. (SAV)

## Abbreviations:

PCDD: polychlorinated dibenzo-p-dioxins
PCDF: polychlorinated dibenzofurans
TCDD: 2,3,7,8-Tetrachlorodibenzo-p-dioxin
PCB: polychlorinated biphenyls
PBDE: polybrominated diphenylethers
AhR: aryl hydrocarbon receptor
BMI: body mass index
HR: high resolution
GC/MS: gas chromatography/mass spectrometry
HbA1c: hemoglobin A1c; glycosylated hemoglobin
DDE: dichlorodiphenyldichloroethene
DDT: dichlorodiphenyltrichloroethane
PBB: polybrominated biphenyls
I-TEQ: International Toxic Equivalents
dl: dioxin-like
RA: retinoic acid
